# Supplementary material for: Prediction of designer-recombinases for DNA editing with generative deep learning
Source: Nat Commun. 2022 Dec 27;13:7966. doi: 10.1038/s41467-022-35614-6 (PMC9794738; doi:10.1038/s41467-022-35614-6)
Supplement: Supplementary file 2 — Reporting Summary [file 41467_2022_35614_MOESM2_ESM.pdf]

nature portfolio

Corresponding author(s): Frank Buchholz

Last updated by author(s): Nov 23, 2022

Reporting Summary

Nature Portfolio wishes to improve the reproducibility of the work that we publish. This form provides structure for consistency and transparency in reporting. For further information on Nature Portfolio policies, see our Editorial Policies and the Editorial Policy Checklist.

Statistics

For all statistical analyses, confirm that the following items are present in the figure legend, table legend, main text, or Methods section.

n/a	Confirmed	
☐	☒	The exact sample size (*n*) for each experimental group/condition, given as a discrete number and unit of measurement
☒	☐	A statement on whether measurements were taken from distinct samples or whether the same sample was measured repeatedly
☒	☐	The statistical test(s) used AND whether they are one- or two-sided<br>*Only common tests should be described solely by name; describe more complex techniques in the Methods section.*
☒	☐	A description of all covariates tested
☒	☐	A description of any assumptions or corrections, such as tests of normality and adjustment for multiple comparisons
☐	☒	A full description of the statistical parameters including central tendency (e.g. means) or other basic estimates (e.g. regression coefficient) AND variation (e.g. standard deviation) or associated estimates of uncertainty (e.g. confidence intervals)
☒	☐	For null hypothesis testing, the test statistic (e.g. *F*, *t*, *r*) with confidence intervals, effect sizes, degrees of freedom and *P* value noted<br>*Give P values as exact values whenever suitable.*
☒	☐	For Bayesian analysis, information on the choice of priors and Markov chain Monte Carlo settings
☒	☐	For hierarchical and complex designs, identification of the appropriate level for tests and full reporting of outcomes
☐	☒	Estimates of effect sizes (e.g. Cohen's *d*, Pearson's *r*), indicating how they were calculated

Our web collection on statistics for biologists contains articles on many of the points above.

Software and code

Policy information about availability of computer code

Data collection	Recombinase sequence data used to train the generative model was processed with PacBio's ccs v3.4.1, SAMtools v1.11, exonerate v2.3.0, GNU grep v3.7, GNU Awk v5.1.1, GNU sed v4.8.
Data analysis	Recombinase sequence data analysis was performed with R v4.1.1 with tidyverse v1.3.1, ggplot 3.3.3, Rtsne v0.1.5, and stringdist v0.9.8. Neural networks for generative models were trained using Python v3.9.6 with numpy v1.22.1, pandas v1.4.0 and pytorch v1.10.1. Custom scripts for the neural networks can be found at https://github.com/ltschmitt/RecGen.

For manuscripts utilizing custom algorithms or software that are central to the research but not yet described in published literature, software must be made available to editors and reviewers. We strongly encourage code deposition in a community repository (e.g. GitHub). See the Nature Portfolio guidelines for submitting code & software for further information.

Data

Policy information about availability of data

All manuscripts must include a data availability statement. This statement should provide the following information, where applicable:

- Accession codes, unique identifiers, or web links for publicly available datasets
- A description of any restrictions on data availability
- For clinical datasets or third party data, please ensure that the statement adheres to our policy

The sequence data generated in this study from published evolution campaigns have been deposited in the European Nucleotide Archive under accession code PRJEB57361. The sequence data generated in this study from non-published evolution campaigns are available under restricted access for intellectual property

reasons, access can be obtained by contacting the corresponding author F.B. on reasonable request. Source data are provided with this paper.

Human research participants

Policy information about studies involving human research participants and Sex and Gender in Research.

Reporting on sex and gender	N/A
Population characteristics	N/A
Recruitment	N/A
Ethics oversight	N/A

Note that full information on the approval of the study protocol must also be provided in the manuscript.

Field-specific reporting

Please select the one below that is the best fit for your research. If you are not sure, read the appropriate sections before making your selection.

[x] Life sciences [ ] Behavioural & social sciences [ ] Ecological, evolutionary & environmental sciences

For a reference copy of the document with all sections, see nature.com/documents/nr-reporting-summary-flat.pdf

Life sciences study design

All studies must disclose on these points even when the disclosure is negative.

Sample size	The number of sequences used to train the model was determined by the size of the library with the least amount of reads. Additionally, we found that decreasing the amount of sequences did not improve performance of the algorithm.
Data exclusions	To maintain equal ratios between the data input we had to reduce the amount of sequences we used for our algorithm. We therefore excluded sequences that were beyond the determined sequence amount.
Replication	The generative algorithm training was performed multiple times to generate multiple models. All models were used for prediction. The amount of models generated is indicated in the Methods section. Experimental validation of recombinase activity was not replicated, because they were independently validated with additional assays.
Randomization	Input data order was randomized during training.
Blinding	No blinding was performed, as sequence validation of the experiments delivers undeniable proof of recombination.

Reporting for specific materials, systems and methods

We require information from authors about some types of materials, experimental systems and methods used in many studies. Here, indicate whether each material, system or method listed is relevant to your study. If you are not sure if a list item applies to your research, read the appropriate section before selecting a response.

Materials & experimental systems

n/a	Involved in the study
[x]	[ ] Antibodies
[x]	[ ] Eukaryotic cell lines
[x]	[ ] Palaeontology and archaeology
[x]	[ ] Animals and other organisms
[x]	[ ] Clinical data
[x]	[ ] Dual use research of concern

Methods

n/a	Involved in the study
[x]	[ ] ChIP-seq
[x]	[ ] Flow cytometry
[x]	[ ] MRI-based neuroimaging
