# Supplementary material for: Prediction of designer-recombinases for DNA editing with generative deep learning
Source: Nat Commun. 2022 Dec 27;13:7966. doi: 10.1038/s41467-022-35614-6 (PMC9794738; doi:10.1038/s41467-022-35614-6)
Supplement: Supplementary file 3 — Description of Additional Supplementary Files [file 41467_2022_35614_MOESM3_ESM.pdf]

Title: Supplementary Data 1:
Description: Target site names, their associated publications and the previously published name.

Title: Supplementary Data 2:
Description: Positions of observed residue changes in comparison to Cre. Reference to the publications where changes on these positions where mentioned before. Percentages indicate the amount of sequences that were found with a change compared to Cre.

Title: Supplementary Data 3:
Description: Predicted recombinases used for experimental validation. Includes the target site sequences, protein sequences and gene sequences.
